# Post-ischemic enteritis mimicking Crohn's disease with complex entero-enteric fistulization

**DOI:** 10.1016/j.radcr.2026.09.071

**Published:** 2026-10-24

**Authors:** Faeze Salahshour, Sahar Yaghoubi, Nasim Khajavirad, Amir Ghasemloee

**Affiliations:** a Department of Radiology, Advanced Diagnostic and Interventional Radiology Research Centre, Imam Khomeini Hospital Complex, Tehran University of Medical Sciences, Tehran, Iran; b Department of Internal Medicine, Imam Khomeini Hospital Complex, Tehran University of Medical Sciences, Tehran, Iran; c Department of Surgery, Imam Khomeini Hospital Complex, Tehran University of Medical Sciences, Tehran, Iran

**Keywords:** Mesenteric venous thrombosis, Crohn's disease mimic, Ischemic enteritis, Bowel fistulization, Small bowel obstruction, Chronic mesenteric ischemia

## Abstract

Mesenteric venous thrombosis is an uncommon yet potentially life-threatening cause of mesenteric ischemia with an insidious clinical course. Delayed diagnosis of venous ischemia may lead to late complications such as bowel strictures, perforation, and fistulization. These sequelae can closely mimic Crohn's disease, posing significant diagnostic and therapeutic challenges.

We report the case of a 33-year-old postpartum woman who presented with acute abdominal pain and was diagnosed with subacute thrombosis of the superior mesenteric vein and portal vein confluence on contrast-enhanced CT. Initial imaging demonstrated jejunal wall thickening without signs of transmural necrosis, and the patient was managed conservatively with anticoagulation. Despite treatment, she later developed recurrent abdominal pain, bowel obstruction, perforation, and complex small bowel fistulization. Serial imaging findings were initially interpreted as fibrostenotic Crohn's disease. Endoscopic evaluation and bowel biopsies were unremarkable. Ongoing recurrent obstruction ultimately necessitated limited small bowel resection, and histopathologic findings confirmed ischemic injury, characterized by ulceration, vascular congestion, granulation tissue, and full-thickness hyalinization and necrosis. The patient unfortunately died from postoperative complications seven months after the initial presentation.

This case highlights chronic ischemic enteritis as an important masquerader of Crohn's disease, particularly in patients with a history of mesenteric venous thrombosis. Complex fistulization and fibrostenotic changes are not pathognomonic for inflammatory bowel disease and may represent late sequelae of missed or patchy bowel ischemia. Careful assessment of mesenteric vasculature and collateral formation on imaging is crucial to avoid misdiagnosis, inappropriate immunosuppressive therapy, and delayed definitive management.

## Introduction

Mesenteric ischemia, though rare, poses a significant threat to life. Ischemia due to venous thrombosis, constituting 5–15% of cases, manifests a particularly insidious diagnostic course [1]. Although bowel necrosis is less frequent than arterial compromise, it can be missed due to vague symptoms, leading to delayed complications such as bowel perforation, peritonitis, bowel strictures, and fistulization [2]. These late sequelae can mimic other conditions, such as Crohn's disease, making accurate diagnosis and management challenging.

## Case presentation

A 33-year-old female presented to the hospital one month postpartum with acute abdominal pain. Physical examination revealed abdominal distension and diffuse tenderness without signs of peritonitis. Initial laboratory tests showed mild leukocytosis (WBC: 15,900 × 103/L, neutrophils: 70%), anemia (Hb: 9.8 g/dL), and normal platelet count (193,000 × 103/L). Renal and hepatic function tests were within normal limits.

Further history displayed three repeated miscarriages in the first and second trimesters with no proven hypercoagulable state. Further coagulation studies revealed: Protein S: 53 MgEq/mL (55–160), Protein C: 83 MgEq/mL (70–140), Antithrombin III activity: 86 MgEq/mL (80–120), INR: 1.52, Anti-Cardiolipin IgG: 1.38 MgEq/mL, Anti-Cardiolipin IgM: 3.99 MgEq/mL, B2 Glycoprotein IgG: 4.2 MgEq/mL, B2 Glycoprotein IgM: 14.9 MgEq/mL. All other rheumatologic evaluations, including C3, C4 complement factors, ANA, Anti-dsDNA, P-ANCA, and C-ANCA, were within normal ranges.

An abdominopelvic CT scan with contrast revealed subacute thrombosis of the portal vein confluence and superior mesenteric vein (SMV), with collateral formation in the porta hepatis and mesenteric root (Fig. 1). Mild ascites, bowel distension, and wall thickening were noted in the jejunum, suggestive of bowel ischemia (Fig. 2). However, there was no evidence of transmural necrosis, pneumatosis, or perforation. The patient was managed conservatively with anticoagulants.

**Fig. 1:**
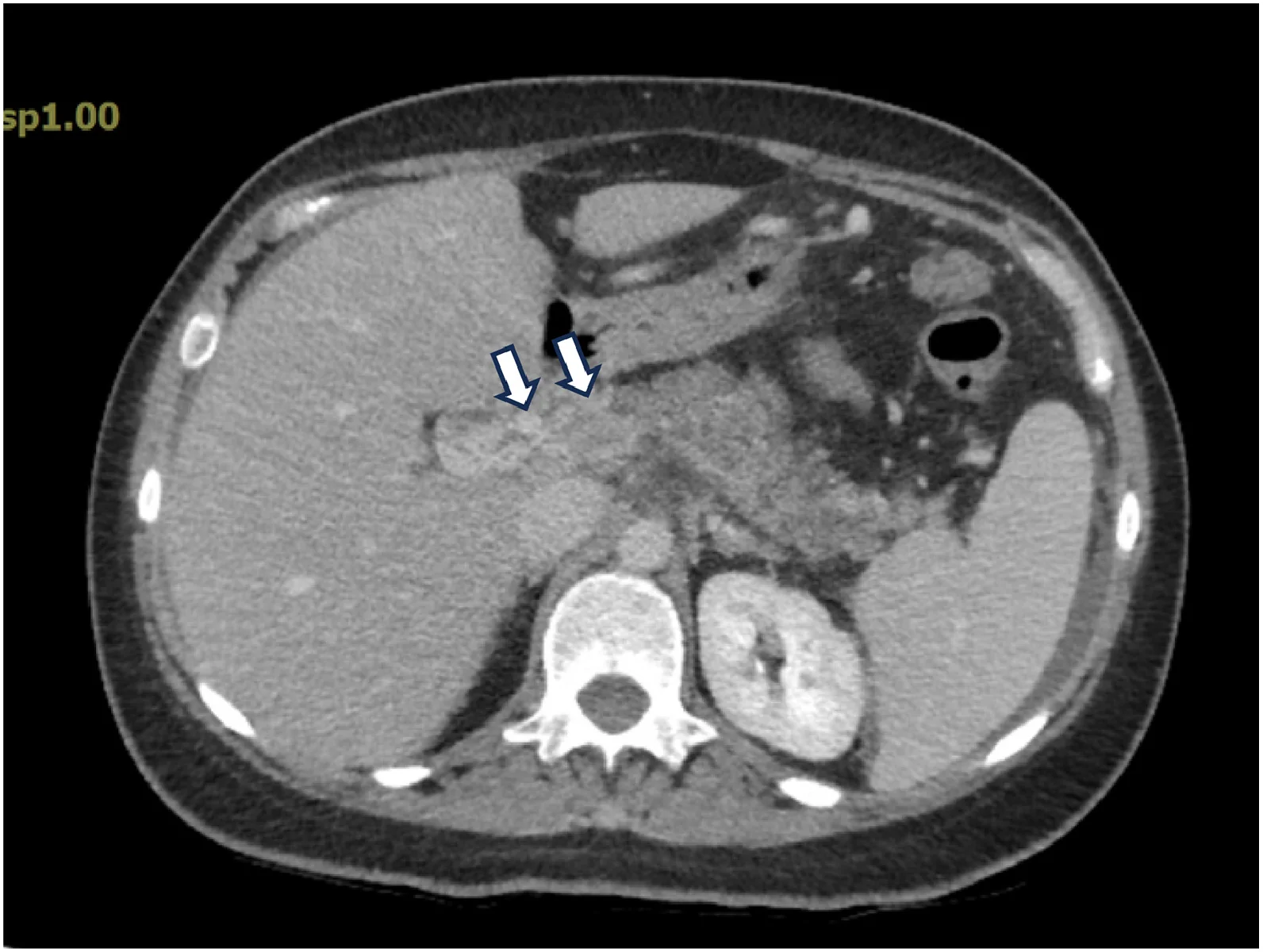
Contrast-enhanced axial CT image demonstrates thrombosis of the portomesenteric venous confluence with development of collateral venous channels in the porta hepatis (white arrow), indicating established alternative venous drainage in the setting of subacute mesenteric venous thrombosis.

**Fig. 2:**
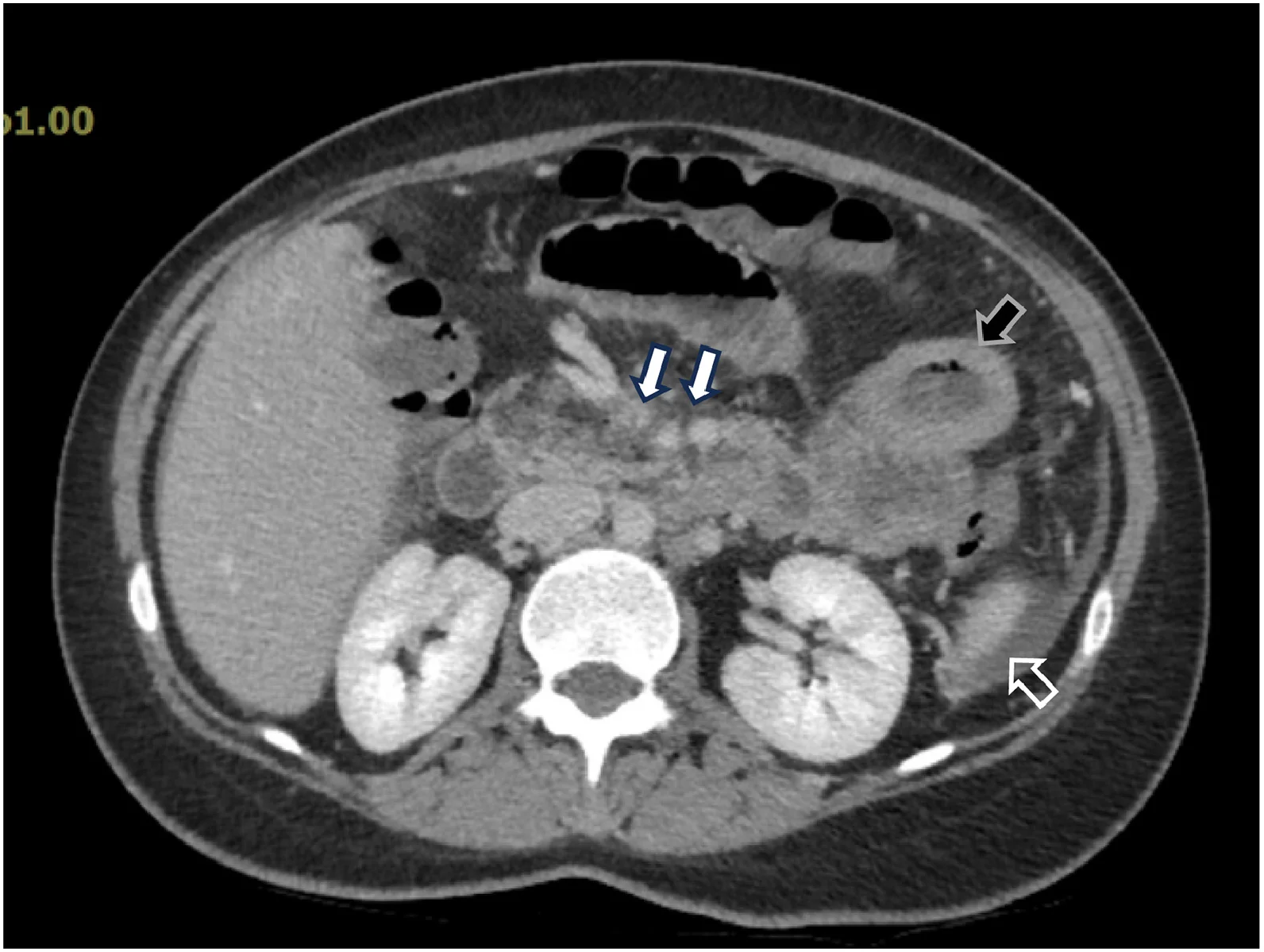
Contrast-enhanced axial CT image demonstrates prominent collateral venous channels within the mesenteric root (solid white arrow), associated with circumferential mural thickening and mild dilatation of a jejunal loop (black arrow) and adjacent ascites (open white arrow). These findings are consistent with venous congestion and bowel ischemia in the setting of mesenteric venous thrombosis.

A follow-up CT scan two weeks later showed a lack of mural enhancement on the mesenteric side of several jejunal loops, with preserved antimesenteric enhancement. No free air or extraluminal gas was observed. Considering the extent of involvement and no signs of overt peritonitis on physical exam, conservative management was continued, and the patient was discharged on direct oral anticoagulants (DOACs).

Two months later, the patient was readmitted with severe abdominal pain and signs of peritonitis. CT demonstrated pneumoperitoneum secondary to bowel perforation, bowel obstruction, and complex fistulization between bowel loops that had previously demonstrated mesenteric-sided ischemic changes (Fig. 3, Fig. 4, Fig. 5). A new 2 cm right atrial thrombus was also identified (Fig. 6). Surgical exploration revealed pale, fibrotic, densely adherent, and matted ischemic bowel loops with complex interloop fistulization. Although perforation and fistulization were present, the perforation appeared sealed, and the extensive length of involved bowel meant that resection would have required substantial small-bowel removal, with a high risk of short-bowel syndrome. Immediate bowel resection was therefore deferred, and peritoneal lavage was performed. Following improvement in abdominal pain and overall clinical condition, the patient was discharged.

**Fig. 3:**
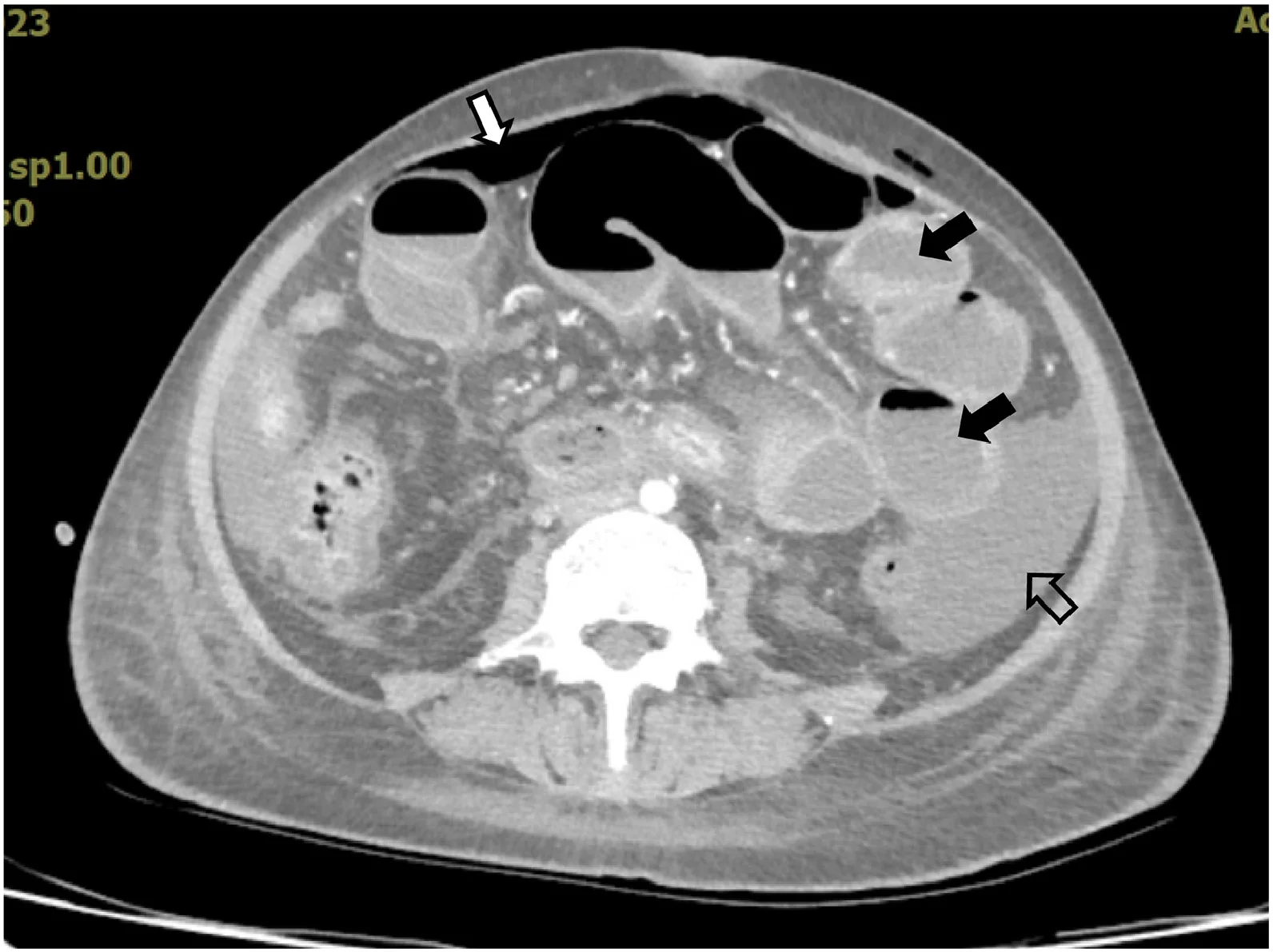
Follow-up contrast-enhanced axial CT image obtained after clinical deterioration demonstrates diffuse small-bowel dilatation consistent with obstruction (solid black arrows), associated with pneumoperitoneum (white arrow) and moderate ascites (open black arrow), indicating interval bowel perforation.

**Fig. 4:**
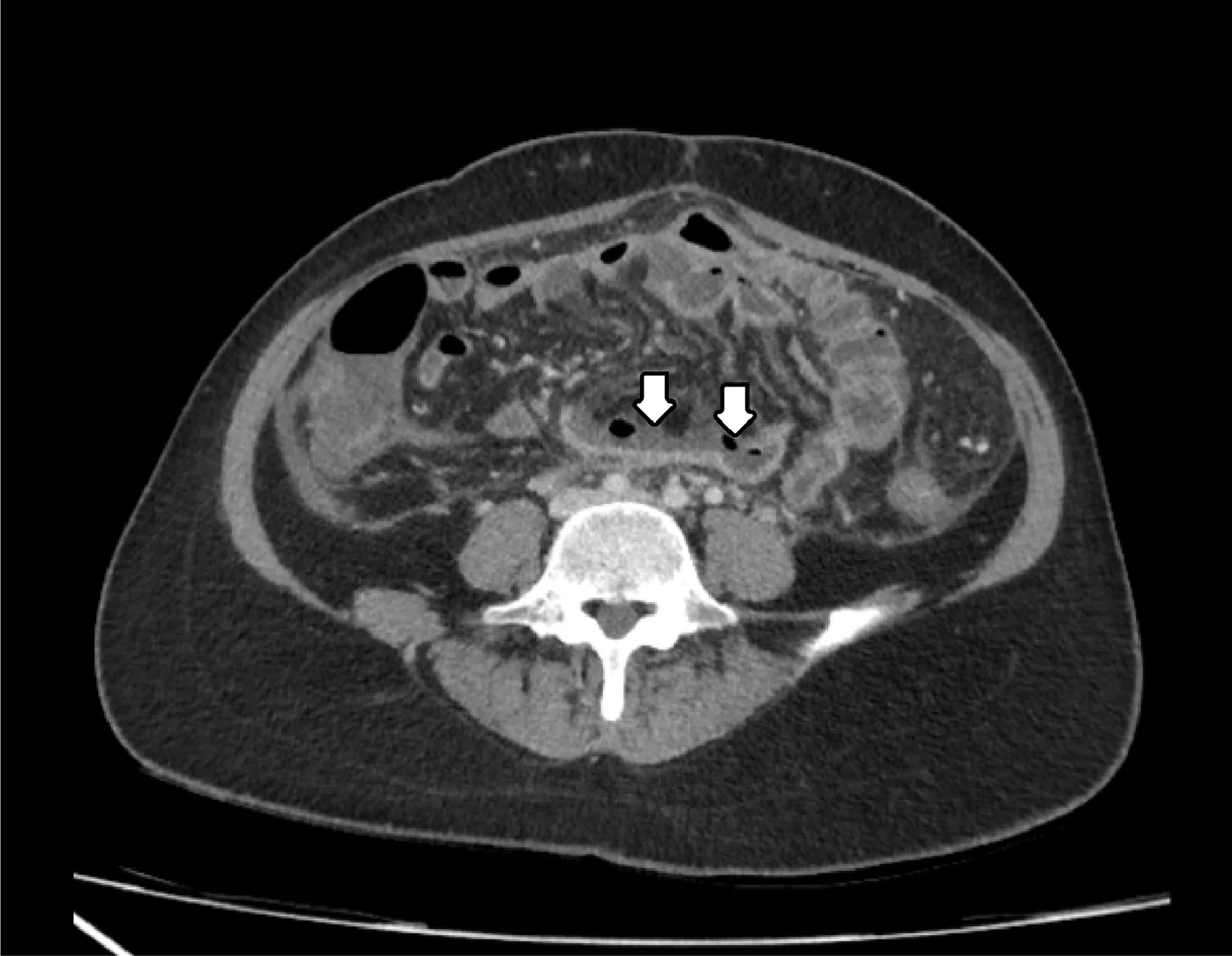
Contrast-enhanced axial CT image demonstrates focal loss of mural integrity and markedly reduced/absent enhancement along the mesenteric aspect of the involved jejunal loop (white arrows), raising concern for advanced ischemic bowel injury.

**Fig. 5:**
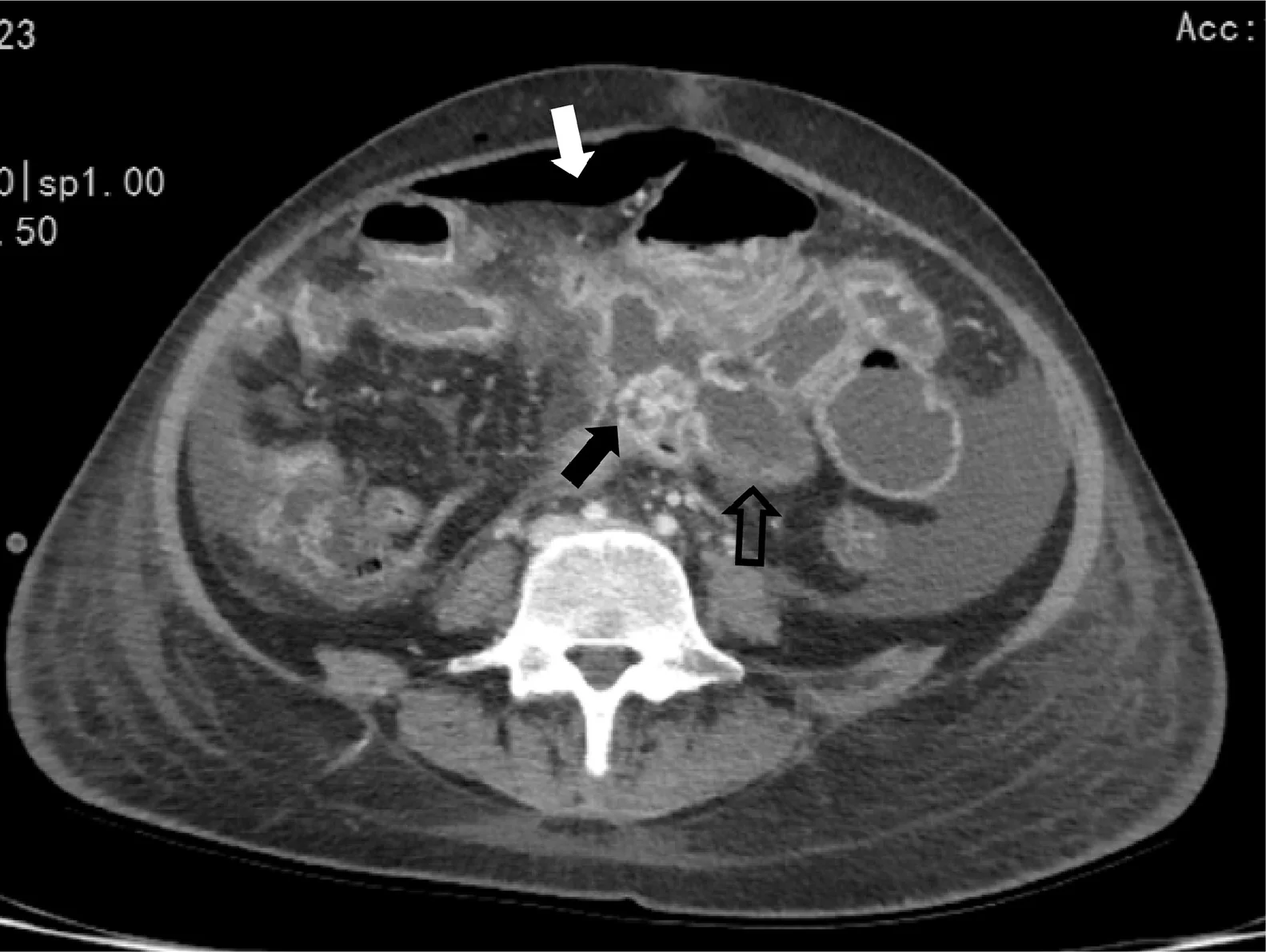
Contrast-enhanced axial CT image demonstrates complex fistulous communication between adjacent jejunal loops (solid black arrows), associated with focal loss of bowel-wall integrity (open black arrow) and pneumoperitoneum (solid white arrow). These findings demonstrate progression of the previously ischemic bowel segments to perforation and complex interloop fistulization.

**Fig. 6:**
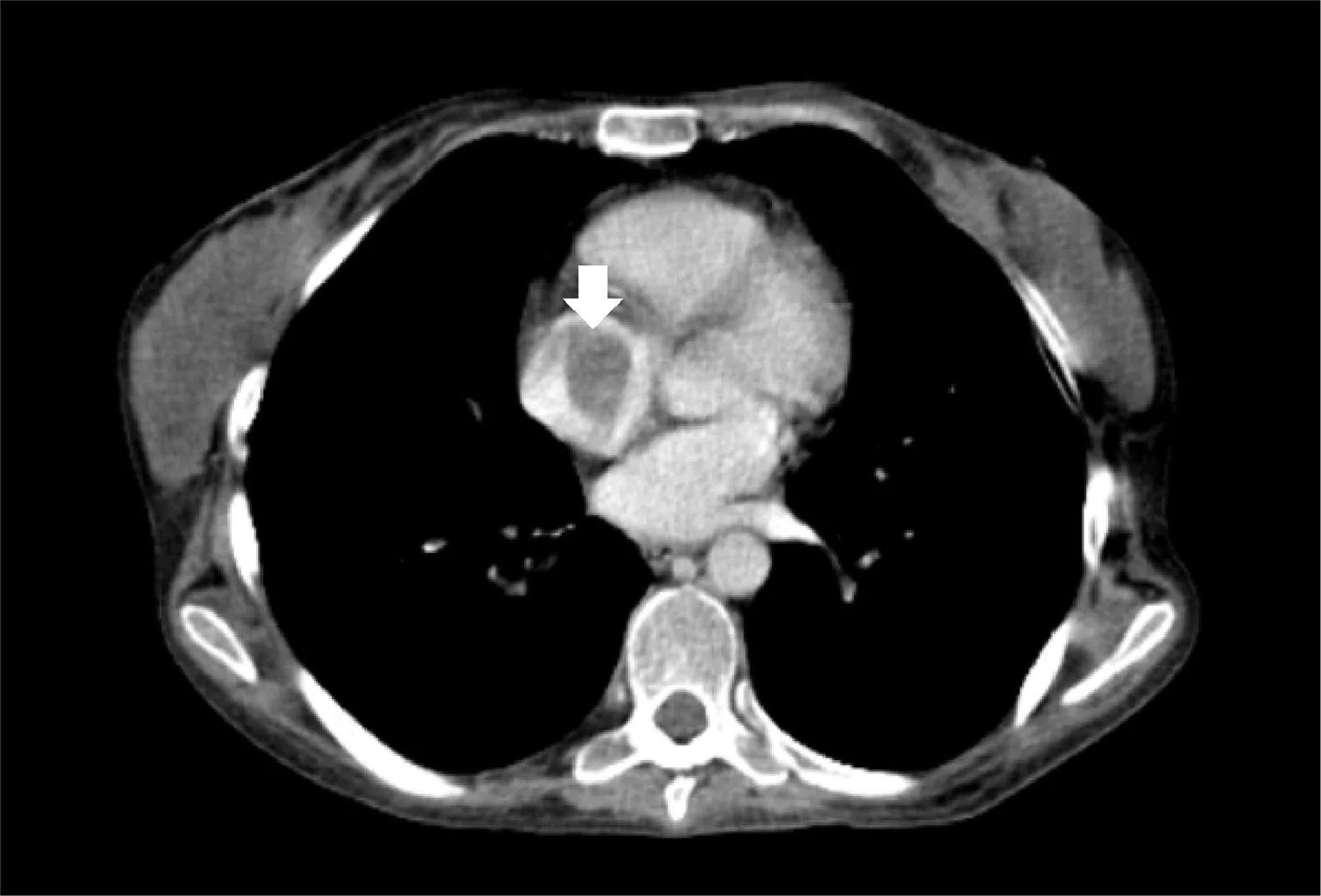
Contrast-enhanced axial CT image through the chest demonstrates a 2 cm filling defect within the right atrium (white arrow), consistent with a right atrial thrombus that developed during follow-up.

A subsequent colonoscopy was performed to exclude inflammatory bowel disease (IBD), with no significant macroscopic or histopathological abnormalities identified in the colonic mucosal biopsies.

Although the right atrial thrombus remained stable in size, the patient experienced recurrent episodes of bowel obstruction every 2–3 months, with serial imaging repeatedly raising the possibility of fibrostenotic Crohn’s disease with complex fistulization. Ultimately, a 20 cm segment of small bowel was resected to relieve the recurrent obstruction. Histopathological examination of the resected specimen confirmed the ischemic nature of the stricture, demonstrating mucosal ulceration, congested ectatic vessels, granulation tissue, and full- thickness hyalinization and necrosis. The patient subsequently died from postoperative complications seven months after the initial presentation.

## Discussion

Mesenteric venous thrombosis (MVT) accounts for approximately 5–15% of cases of mesenteric ischemia and predominantly involves the superior mesenteric vein (SMV) [1,3]. MVT may be primary when no underlying cause is identified or secondary to a predisposing condition. Based on the development of collateral venous circulation, MVT may follow an acute, subacute, or chronic course. In subacute and chronic thrombosis, collateral pathways may provide alternative routes for venous drainage and thereby reduce the likelihood of extensive bowel infarction [2,4].

From a radiological standpoint, CT findings of mesenteric venous ischemia reflect the degree of impaired venous outflow and commonly include circumferential bowel-wall thickening secondary to submucosal edema or hemorrhage, which may produce a stratified “target” or “halo” appearance. Mural enhancement varies with the severity and stage of ischemia, ranging from hyperenhancement related to venous congestion or reperfusion to reduced or absent enhancement as perfusion becomes severely compromised [5,6]. Associated findings include mesenteric venous engorgement, mesenteric edema or fat stranding, ascites, direct visualization of thrombus within the mesenteric or portal veins, and, particularly in subacute or chronic thrombosis, development of collateral venous pathways [6,7]. Persistent venous congestion and ischemic injury may subsequently result in mural fibrosis, luminal narrowing, obstruction, and, less commonly, fistulization [7,8].

Differentiating chronic ischemic enteritis from Crohn’s disease can be challenging because both conditions may demonstrate bowel-wall thickening, stratified enhancement, strictures, fibrosis, and penetrating complications. Crohn’s disease, however, more typically demonstrates asymmetric mural thickening and hyperenhancement, often predominantly along the mesenteric border, engorgement of the vasa recta (the “comb sign”), and fibrofatty proliferation (“creeping fat”), whereas identification of mesenteric venous thrombosis and collateral venous pathways favors a vascular etiology [[5], [6], [9]]. Chronic ischemic injury may also produce segmental ulceration with intervening relatively preserved mucosa, further contributing to its resemblance to Crohn’s disease [10].

In our patient, SMV and portal confluence thrombosis with extensive collateral formation, followed by asymmetric loss of mural enhancement along the mesenteric aspect of several jejunal loops, provided important evidence of an ischemic rather than primarily inflammatory process. Subsequently, the same previously ischemic bowel segments developed perforation, and complex fistulization. In retrospect, the persistence of the portomesenteric collateral network on subsequent imaging, together with the temporal relationship between the initial vascular event and later stricturing and fistulization, should have prompted reconsideration of the presumptive diagnosis of fibrostenotic Crohn’s disease.

CT can strongly suggest, but cannot by itself establish, transmural bowel necrosis. Findings such as absent or markedly reduced mural enhancement, pneumatosis intestinalis, portomesenteric venous gas, and focal bowel-wall discontinuity may raise concern for advanced or transmural ischemic injury; however, definitive confirmation of bowel necrosis requires surgical and/or histopathological assessment [5,6,11,12]. In our patient, asymmetric mesenteric-sided loss of mural enhancement on the second CT was concerning for progressive severe ischemic injury. The ischemic nature of the subsequent stricture was ultimately confirmed histopathologically, with mucosal ulceration, congested ectatic vessels, granulation tissue, and full-thickness hyalinization and necrosis.

Anticoagulation remains the mainstay of treatment for MVT in the absence of clinical or imaging findings requiring urgent surgical intervention. Nevertheless, the insidious clinical course of venous ischemia may result in delayed recognition of severe bowel injury. Persistent or previous ischemic injury can evolve into fibrosis and post-ischemic stenosis, presenting weeks to months after the initial thrombotic event and causing recurrent bowel obstruction [2,7,8]. Perforation may similarly present acutely with peritonitis or remain contained, potentially resulting in abscess formation or fistulization. Although fistulization is strongly associated with penetrating Crohn’s disease, ischemic enteroenteric or enterocolic fistulas have also been reported [13]. Thus, the combination of stricture and complex fistulization should not by itself be regarded as diagnostic of Crohn’s disease.

The differential diagnosis of small-bowel wall thickening, strictures, and fistulization extends beyond Crohn’s disease and chronic ischemia. Intestinal tuberculosis, radiation enteritis, vasculitis, NSAID-related enteropathy, and small-bowel malignancy, particularly lymphoma, should also be considered according to the clinical setting and imaging distribution. Intestinal tuberculosis commonly involves the ileocecal region and may produce mural thickening, strictures, lymphadenopathy, and, less commonly, fistulization [14]. Chronic radiation enteritis may manifest as bowel-wall thickening, fibrosis, strictures, or fistulas within previously irradiated bowel segments [14]. NSAID enteropathy is characterized particularly by multiple short-segment, symmetric circumferential strictures or diaphragm-like narrowing and should be considered in the appropriate medication history [15]. Careful assessment of the distribution and morphology of bowel abnormalities, mesenteric vasculature, collateral circulation, and relevant clinical history is therefore essential when differentiating these entities.

This case illustrates how chronic post-ischemic bowel changes may evolve into a morphological pattern closely resembling fibrostenotic and penetrating Crohn’s disease. Although post-ischemic jejunal stenosis and ischemic fistulization are uncommon, both have previously been reported [7,13]. In patients with a history of mesenteric venous thrombosis, correlation with earlier imaging and careful reassessment of the mesenteric vasculature and collateral pathways are particularly important when new strictures, obstruction, or fistulas develop [7,8,13].

## Conclusion

This case report highlights the diagnostic challenges, as ischemic enteritis or colitis may be unresponsive to biologics or immunomodulators used in Crohn's, instead requiring revascularization, anticoagulation, or surgery. Clinicians should prioritize vascular imaging (eg, CT angiography/venography) in patients with risk factors for thrombosis or ischemia, especially when endoscopic or radiographic findings suggest fibrostenotic disease without clear inflammatory markers. Early recognition prevents unnecessary immunosuppressive exposure and addresses the underlying vascular pathology, improving outcomes in these masquerading cases.

## Author contributions

All authors contributed to the study conception and design. Material preparation, data collection, and analysis were performed by Faeze Salahshoor and Sahar Yaghoubi. Clinical management was provided by Nasim Khajavirad and Amir Ghasemloee. The first draft of the manuscript was written by Sahar Yaghoubi, and all authors commented on previous versions of the manuscript. All authors read and approved the final manuscript.

## Patient consent

Written informed consent was obtained from the patient/legal guardians for the publication of this case report, including any accompanying images.
